# Oxygenated Volatile Organic Compounds (Anti-freezing Agents) in Decorative Water-based Paints Marketed in Nigeria

**DOI:** 10.5696/2156-9614-8.18.180606

**Published:** 2018-06-11

**Authors:** Ajoke F. Idayat Apanpa-Qasim, Adebola A. Adeyi

**Affiliations:** 1 Department of Chemistry, University of Ibadan, Ibadan-Nigeria; 2 CSIR-National Environmental Engineering and Research Institute, Nagpur, India

**Keywords:** water-based paints, anti-freezing agents, human exposure, glycols, volatile organic compounds

## Abstract

**Background.:**

Consumer products such as paints are a potentially significant source of volatile organic compounds (VOCs) and oxygenated VOCs. Paints for construction and household use have been rapidly changing from oil-based to water-based paints and are one of the commonly identified sources of oxygenated VOCs in indoor environments.

**Objectives.:**

Four different anti-freezing agents were identified and analyzed in 174 waterbased paint samples, purchased from popular paint markets in two metropolitan cities in Nigeria, Lagos and Ibadan.

**Methods.:**

Paint samples were solvent extracted using acetonitrile and milli-Q water. Antifreezing agents in the extracts were identified and quantified using gas chromatography (GC)-mass spectrometry and a GC-flame ionization detector, respectively.

**Discussion.:**

Four different anti-freezing agents were identified in the samples, ethylene glycol, diethylene glycol, triethylene glycol and propylene glycol. Their levels ranged from 1,000-1,980 ppm, diethylene glycol; 1,000–3,900 ppm, triethylene glycol; 1,090–2,510 ppm, propylene glycol and 1,350–2,710 ppm, ethylene glycol. Levels of anti-freezing agents in all of the paint samples were above the permissible limits of the European Union for VOCs in paints of 500 ppm. Results of multivariate statistical analyses clearly showed that triethylene glycol was the most commonly used anti-freezing agent in paints despite its numerous harmful health effects.

**Conclusions.:**

We concluded that water-based paints marketed in Nigeria contain high concentrations of anti-freezing agents, which have harmful environmental and human health effects, especially to sensitive individuals such as children.

**Competing Interests.:**

The authors declare no competing financial interests.

## Introduction

Volatile organic compounds (VOCs) and oxygenated VOCs such as alcohols, aldehydes, ketones, phenols, esters, ethers, carboxylic acids and their derivatives are considered to be priority pollutants of the atmosphere and aquatic environment.^1^ They are reported to have carcinogenic and mutagenic properties, a high degree of ecotoxicity and also play a significant role in the formation of secondary air pollutants, such as tropospheric ozone.^2–5^ Anti-freezing agents such as propylene glycol, ethylene glycol, diethylene glycol and triethylene glycol are important oxygenated VOCs. They are used in paints for flow control and application properties, stability while in the liquid state and suitability to all weather conditions.^6^ Anti-freezing agents not only irritate the eyes and skin, but also affect the kidney and central nervous system, sometimes inducing renal failure and brain injury.^7^,^8^ The European Union has set 500 ppm as the permissible limit of VOCs in low emission paints, while ethylene glycol and diethylene glycol are prohibited in paints.^9^ Presently, there are no national regulations controlling anti-freezing agents in paints in Nigeria.

Propylene glycol (1, 2-propanediol) is an organic compound with multiple uses including as a humectant, food additive, drug solvent and a moisturizer in medicines, cosmetics, and tobacco products. Propylene glycol has been used as a carrier in fragrance oils, non-toxic antifreeze for winterizing drinking water systems, paints production, as a coolant in liquid cooling systems and is the main ingredient in deodorant sticks.^10^,^11^ Ethylene glycol is a colorless, practically odorless organic compound whose reactivity and solubility provide the basis for many applications, including solvent coupler, freezing point depression in water-based formulations, solvent and humectant.^12^ Triethylene glycol is a transparent, colorless, low-volatility, moderate-viscosity, water soluble liquid. It has properties similar to other glycols and may be used preferentially in applications requiring a higher boiling point, higher molecular weight, or lower volatility than diethylene glycol.^13^ It may be used directly as a plasticizer or modified by esterification. The solubility property of triethylene glycol is important for many applications. End-uses of triethylene glycol are numerous; including hygroscopicity, gas dehydration, as a solvent and for freezing point depression.^14^ Diethylene glycol is a colorless, low-volatility, low viscosity, and hygroscopic liquid. Because of its higher molecular weight, diethylene glycol is considerably less volatile than ethylene glycol and differs sufficiently in that it has specialized uses. It is used for hygroscopicity, as a lubricant, solvent coupler (stabilizer for soluble oil dispersions), compatibilizer for dye and printing ink components, as a solvent in aromatic and paraffinic hydrocarbon separations, printing ink/paint pigment/dye, for freezing point depression in latex paint, deicing fluid and heat transfer fluid.^15^

Changes in building designs to improve energy efficiency have meant that modern homes and offices are frequently more airtight than older structures. Advances in construction technology have also led to much greater use of synthetic building materials. While these improvements have led to more comfortable buildings, they also lead to indoor environments in which contaminants, VOCs and oxygenated VOCs such as anti-freezing agents are readily produced and may build-up to much higher concentrations than are found outside.^16^ Previous studies on VOCs emitted by consumables in indoor environments include burning of candles, tobacco smoke, as well as cooking, heating, and office equipment, paint and associated supplies, adhesive, detergent, wax, furnishing, clothing, building materials, combustion materials, and appliances.^17–29^ The present study identifies and assesses the levels of anti-freezing agents used in water-based paints sold in Nigeria and their associated health effects. It also compares anti-freezing agent levels in paints with the available permissible limit and threshold limit. Data were interpreted using multivariate statistical analysis.

**Abbreviations**
*PCA*: Principal component analysis

## Methods

### Paint sample collection

Water-based paint samples were purchased in popular paint markets in Ibadan and Lagos, Nigeria, based on color availability and those most commonly used as presented in Table 1. A total of 174 paint samples from 14 different manufacturers were collected. Some of the products purchased were imported by manufacturer C, but their country of production was not disclosed by the paint vendor and retailer at the point of purchase, thus their origin was not known. Samples were stored in airtight plastic containers, transported and analyzed at the Council of Scientific and Industrial Research-National Environmental Engineering Research Institute Laboratory, Nagpur – Maharashtra, India.


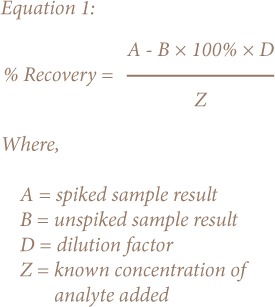


**Table 1:** Paint Samples Collected in Lagos and Ibadan Metropolises, Nigeria

**Serial No.**	**Manufacturer codes**	**Number of different paint colors collected**	**Number of paint samples collected**	**NIS/ISO registration**
1	A	10	20	Available
2	B	9	18	Available
3	C	9	18	Available
4	D	5	10	Available
5	E	5	10	Available
6	F	6	12	Available
7	G	4	8	Not available
8	H	5	10	Not available
9	I	4	8	Not available
10	J	5	10	Not available
11	K	6	12	Not available
12	L	8	16	Not available
13	M	7	14	Not available
14	N	4	8	Not available

Abbreviations: ISO, International Organization for Standardization; NIS, Nigerian Industrial Standard

Note: Number of paint colors collected per manufacturer was based on availability

### Sample pre-treatment and analysis

Approximately 2.5 mL of paint samples were carefully measured into 50 mL polypropylene radiation sterilized centrifuge tubes and extracted using liquid-liquid extraction with milli-Q water followed by acetonitrile in the ratio 3:4 (v/v). The centrifuge tubes were shaken and mixed on a cyclo-mixer at 50 cycles (CM 101) for sample homogeneity. The tubes were centrifuged at 5000 rpm at 20°C for 20 minutes. After phase separation, the acetonitrile phase was dehydrated with 1.0 g of sodium sulphate, filtered using a glass micro-fiber filter of 90 mm diameter and stored in 2 mL vials. The aqueous phase was filtered using polytetrafluoroethylene micro-fiber syringe filter of 13 mm diameter and 0.22-micron pore size, and stored in 2 mL Maxipense plastic vials prior to analysis. The acetonitrile phase was identified using gas chromatography (GC)-mass spectrometry while the aqueous phase was quantified using a gas chromatography flame ionization detector (GC-FID).

A recovery study was carried out using 2.5 mL of selected paint samples, which were measured into 50 mL polypropylene sterilized centrifuge tubes and extracted using the liquidliquid extraction described above. Samples were spiked with different concentrations of anti-freezing agents (100–300 ppm) and analyzed using GC-FID. Recovery was calculated using Equation 1. The recovery of anti-freezing agents ranged from 93.5–103% as presented in Table 2.

**Table 2:** Recovery Study of Antifreezing Agents in Paint Samples

**VOCs**	**MS**	**MSD**	**MEAN ±SD**
DEG	87.0	100	93.5±9.2
TEG	105	101	103±3.0
EG	99.9	98.9	99.4±0.7
PG	94.1	96.9	95.5±2.0

Abbreviations: MS, Matrix spike; MSD, Matrix spike duplicate; SD, standard deviation; DEG, diethylene glycol; EG, ethylene glycol; PG, propylene glycol; TEG, triethylene glycol

### Instrument operation conditions

A Perkin Elmer – Clarus 680 GC model with a Perkin Elmer Packard mass spectrometry was used for the identification of various organic components present in the paint samples. Identification was based on the molecular structure, molecular mass and calculated fragments. The column used was Agilent J&W DB-5, a non-polar column (30 m × 0.25 mm × 0.25 μm). Operating conditions were: helium carrier gas with flow rate of 1 mL/minute at atmospheric pressure of 12.9 psi and an initial flow rate of 26.3 cm/second; injection temperature of 200°C; detector temperature of 280°C; and oven temperature program of 60°C for 1.50 minutes and ramped to 280°C at 10°C/minute for 10.00 minutes with a total run time of 33.50 minutes. Electron ionization-mass spectroscopy in selected ion monitoring mode was used for the identification of the anti-freezing agents in the samples (*Table 3*). The spectrum of the unknown compounds was compared with the spectrum of the compounds stored in the Turbomass software and NIST mass spectroscopy search 2.0, 2008 and scanned (10:450) EI+ for identification. The identified compounds along with their retention time, molecular formula, and molecular weight are presented in Table 4.

**Table 3:** Monitoring Ions Used for the Identification of Anti-freezing Agents in Paint Samples

**Anti-freezing agents**	**Monitoring ions (m/z)**
Ethylene glycol	31, 33, 29
Propylene glycol	45, 75, 76
Triethylene glycol	45, 58, 89
Diethylene glycol	45, 75, 43

Note: The underlined number is the m/z of the ion used for quantification

**Table 4:** Compounds Identified in Paint Samples

**Serial No.**	**Retention times (min)**	**NIST nomenclature**	**Molecular weight**	**Molecular formula**	**Class of compounds**
1	2.28	2-Butanone, 3,3-dimethyl	100	C_6_H_12_O	Ketone
2	2.45	Propylene glycol	76.09	C_3_H_8_O_2_	Glycol
3	3.20	Ethylene glycol butyl ether	118.17	C_6_H_14_O_2_	Glycol ether
4	3.50	Ethylene glycol ethyl ether acetate	132.16	C_6_H_12_O_3_	Ester
5	5.36	Diethylene glycol	106.12	C_4_H_10_O_3_	Glycol
6	6.05	Ethylene glycol	62.07	C_2_H_6_O_2_	Glycol
7	6.61	Ethylene glycol butyl ether acetate	160.21	C_8_H_16_O_3_	Ester
8	7.30	Diethylene glycol butyl ether	162.23	C_8_H_18_O_3_	Glycol ether
9	8.71	Triethylene glycol	150.17	C_6_H_14_O_4_	Glycol
10	9.59	2,2,4-trimethyl-1,3-pentane diol diisobut	286	C_16_H_30_O_4_	Texanol
11	9.85	Propanoic acid, 2-methyl-3-hydroxy-2,4	216	C_12_H_24_O_3_	Texanol
12	11.66	Phenol 2,4-Bis (1,1-dimethyl ethyl)-	206	C_14_H_22_O	Phenol
13	12.20	1, Hexadecene	224	C_16_H_32_	Alkene
14	12.45	Furan, tetrahydro-2-methyl	86	C_5_H_10_O	Furan
15	13.30	Dihexyl fumarate	284	C_16_H_28_O_4_	Ester
16	14.38	3 Eicosene	280	C_20_H_40_	Alkene
17	16.32	Dibutyl phthalate	278	C_6_H_22_O_4_	Ester
18	18.05	1, Nonadecene	266	C_19_H_38_	Alkene
19	19.65	11- Tricosene	322	C_23_H_46_	Alkene
20	21.13	Oxalic acid, allyl tetradecyl ester	326	C_19_H_34_O_4_	Ester

A Perkin Elmer – Clarus 500 GC model with Packard FID was used for quantification. An Agilent J&W DB-624 UI, polar column (30 m × 320 μm × 1.80 μm) was used. Ultrapure hydrogen was used as the carrier gas with a flow rate of 45 mL/minute. Operating conditions were: injection temperature of 250°C, detector temperature of 280°C and column temperature of 50°C for 3 minutes ramped to 100°C at 6°C/minute and 250°C at 10°C/minute for 3 minutes with total run time of 29.33 minutes.

### Multivariate statistical analysis

The Statistical Package for the Social Sciences (SPSS Windows version 18) and Excel 2010 software were used for the data analysis. Correlation coefficient, cluster analysis, and principal component analysis (PCA) are statistical tools used for better interpretation of large data.^30^ Cluster analysis is used for dividing the studied parameters into similar classes with respect to their normalized concentration levels. The correlation coefficient shows pair wise association of a set of variables indicating their common source. Principal component analysis is designed to transform the original variables into new, uncorrelated variables (axes), called the principal component, to find the directions (components) that maximize the variance in the dataset.^31^ The outcome of the reduced dimension data set will allow the evaluation of spot trends, patterns and outliers in the data, far more easily than would have been possible without performing PCA. Hence, it is used for source identification, facilitating correlation of associated groups of pollutants or contaminants of interest to their sources.^30^

## Results

### Identification and percentage composition of VOCs and oxygenated VOCs in paint samples

**Figure 1:**
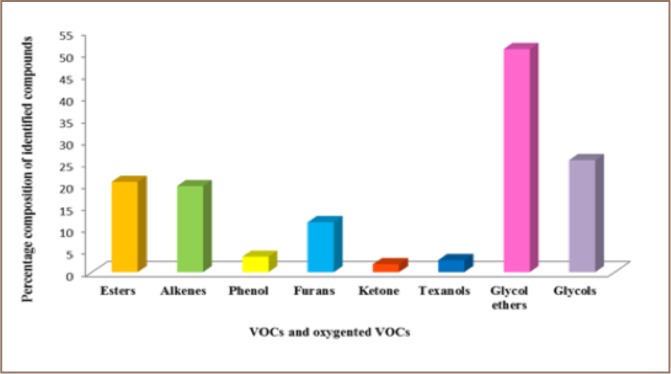
Percentage composition of VOCs and oxygenated VOCs identified in paint samples

The identification of the anti-freezing agents and some other VOCs present in the samples was confirmed based on retention times and molecular formula. Twenty different compounds classified as VOCs and oxygenated VOCs were identified in the samples. They included glycols (19%) which are the anti-freezing agents and include ethylene glycol, diethylene glycol, triethylene glycol, and propylene glycol; glycol ethers (37%); esters (15%) and alkenes (15%). Others are texanol (2%), ketone (1%) and furans (8%) as shown in Figure 1.

### Anti-freezing agent concentrations

**Figure 2:**
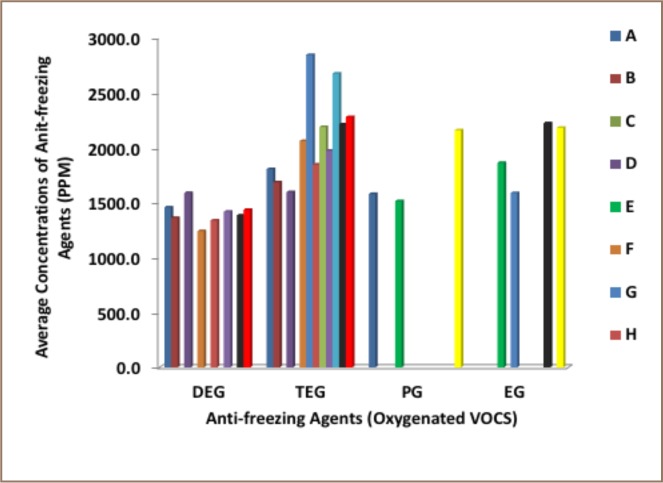
Variation in the concentrations of antifreezing agents in paint samples across manufacturers Abbreviations: DEG, diethylene glycol; EG, ethylene glycol; PG, propylene glycol; TEG, triethylene glycol

Fourteen paint manufacturers were selected: six registered with the Nigerian Industrial Standard (NIS) and International Organization for Standardization (ISO) and eight unregistered manufacturers without NIS and ISO certification, producing different colors of water-based paints. Four anti-freezing agents: ethylene glycol, triethylene glycol, diethylene glycol and propylene glycol were identified in the 174 paint samples collected in Lagos and Ibadan. The antifreezing agents' concentrations across different manufacturers are presented in Supplemental Material 1, while Table 5 shows the mean concentrations of the anti-freezing agents in all of the samples. Variations in the concentrations of the anti-freezing agents with respect to manufacturers are presented in Figure 2.

**Table 5:** Mean Concentrations (±SD) (in ppm) of Anti-freezing Agents in Paint Samples Across Manufacturers

**S/N**	**Manufacturer codes**	**Mean concentrations (ppm)**		**PEG**	**EG**
		**DEG**	**TEG**		
1	A	1,460±45	1,800±95	1,580±98	ND
2	B	1,360±130	1,680±32	ND	ND
3	C	ND	ND	ND	ND
4	D	1,590±91	1,590±36	ND	ND
5	E	ND	ND	1,510±51	1,860±57
6	F	1,240±60	2,060±79	ND	ND
7	G	ND	2,840±130	ND	1,590±68
8	H	1,340±39	1,850±55	ND	ND
9	I	ND	2,190±44	ND	ND
10	J	1,420±32	1,970±78	ND	ND
11	K	ND	2,670±96	ND	ND
12	L	1,390±110	2,200±100	ND	2,220±84
13	M	1,430±64	2,280±97	ND	ND
14	N	ND	ND	2,160±37	2,180±35
**Range**		**ND-1,590**	**ND-2,840**	**ND-2,160**	**ND-2,220**

Abbreviations: DEG, diethylene glycol; EG, ethylene glycol; PG, propylene glycol; TEG, triethylene glycol; ND-not detected; SD, standard deviation; EU permissible limit of VOCs is 500 ppm.

#### Diethylene glycol

The concentrations of diethylene glycol in all of the 174 paint samples ranged from ND, 980 ppm. The highest concentration was 1,980 ppm obtained in paint samples produced by manufacturer F, a registered manufacturer. This was followed by 1,910 ppm and 1,880 ppm in products produced by manufacturers A (a registered manufacturer) and M (an unregistered manufacturer). The highest mean concentration of diethylene glycol was 1,590±91 ppm obtained in paints produced by manufacturer D. This was followed by 1,460±45 ppm and 1,430±64 ppm in paints produced by manufacturers A and M, respectively, while the lowest mean concentration was 1,240±60 ppm in paints produced by manufacturer F. Diethylene glycol was not detected in the paint samples produced by manufacturers C and E (registered manufacturers) and G, I, K, and N (unregistered manufacturers), but was detected in the products produced by the remaining 8 manufacturers.

#### Triethylene glycol

The concentrations of triethylene glycol in the paint samples ranged from ND-3,900 ppm. The highest concentration of triethylene glycol was 3,900 ppm obtained in paint produced by manufacturer G, an unregistered manufacturer. This was followed by 3,850 ppm and 3,550 ppm in paints produced by manufacturers G and K, respectively, (unregistered manufacturers). The highest mean concentration of triethylene glycol was 2,840±130 ppm obtained in paints produced by manufacturer G. This was followed by 2,670±96 ppm and 2,280±97 ppm in paints produced by manufacturer K and M (unregistered manufacturers). The lowest mean concentration was 1,590±36 ppm in paints produced by manufacturer D. Triethylene glycol was absent in the paints produced by manufacturers C, E (both registered manufacturers) and N (an unregistered manufacturer), but present in the products manufactured by the other 11 manufacturers.

#### Propylene glycol

The concentrations of propylene glycol in the paint samples ranged from ND-2,510 ppm. The highest concentration was 2,510 ppm obtained in paint produced by manufacturer N, an unregistered manufacturer. This was followed by 2,460 ppm and 2,350 ppm, also in paints produced by manufacturer N. The highest mean concentration of propylene glycol with respect to manufacturers was 2,160±37 ppm obtained in paints produced by manufacturer N. This was followed by 1,580±98 ppm and 1,510±51 ppm in paints produced by manufacturers A and E, respectively (registered manufacturers). Propylene glycol was not present in any of the paint samples except for those produced by manufacturers A, E and N.

#### Ethylene glycol

The concentrations of ethylene glycol ranged from ND-2,710 ppm. The highest concentration was 2,710 ppm obtained in paint produced by manufacturer L, an unregistered manufacturer. This was followed by 2,700 ppm and 2,520 ppm, in products produced by the same manufacturer. The highest mean concentration of ethylene glycol was 2,220±84 ppm in paints produced by manufacturer L. This was followed by 2,180±35 ppm and 1,860±57 ppm in paints produced by manufacturers N and E, respectively. Ethylene glycol was not detected in any of the paint samples except for those produced by manufacturers E, G, L and N.

### Concentration of anti-freezing agents in paint samples with respect to color

**Figure 3:**
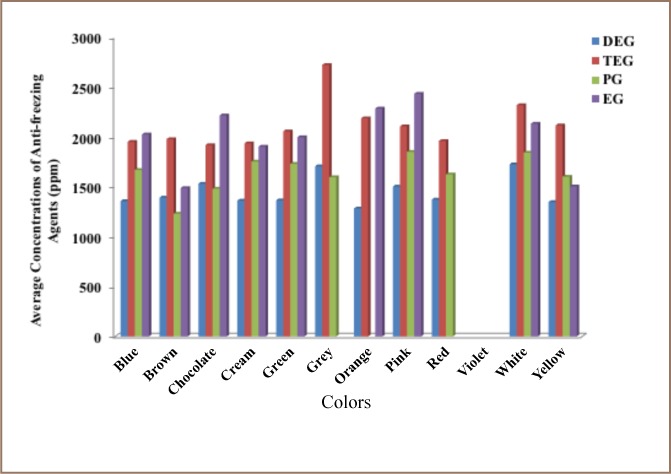
Variation in the concentrations of anti-freezing agents in paints with respect to colors

The concentrations of anti-freezing agents with respect to paint colors are presented in Table 6, while the variation with respect to colors is presented in Figure 3.

**Table 6:** Mean Concentrations (±SD) (in ppm) of Anti-freezing Agents in Paint Samples Across Colors

**Paint color**	**Concentrations (ppm)**			
	**DEG**	**TEG**	**PG**	**EG**
Blue	1,360±93	1,950±100	1,670±23	2,020±100
Brown	1,390±130	1,980±62	1,230±1.7	1,490±70
Chocolate	1,530±77	1,970±29	1,480±40	2,220±45
Cream	1,360±56	1,930±110	1,750±100	1,900±68
Green	1,360±33	2,060±75	1,730±8	2,000±52
Grey	1,700±36	2,720±170	1,600±210	ND
Orange	1,280±100	2,190±67	ND	2,280±4.4
Pink	1,500±95	2,110±31	1,850±74	2,430±120
Red	1,370±150	1,960±98	1,620±28	ND
Violet	ND	ND	ND	ND
White	1,730±220	2,320±81	1,840±140	2,130±4.9
Yellow	1,350±66	2,120±163	1,600±71	1,500±9.0
**Range**	**ND-1,730**	ND-2,720	**ND-1,850**	**ND-2,430**

Abbreviations: DEG, diethylene glycol; EG, ethylene glycol; PG, propylene glycol; TEG, triethylene glycol; ND, not detected; SD, standard deviation

#### Diethylene glycol

The concentrations of diethylene glycol in the paint samples with respect to colors ranged from ND-1,980 ppm. The highest concentration was 1,980 ppm in white paint, followed by 1,910 ppm in cream and 1,880 ppm in white paints. The highest mean concentration of diethylene glycol was 1,730±220 ppm in white paints, followed by 1,700±36 ppm in grey and 1,530±77 ppm in chocolate paints. The lowest concentration, 1,280±100 ppm, was obtained in orange paints. All of the paint colors contained diethylene glycol except violet colored paints.

#### Triethylene glycol

The concentrations of triethylene glycol in the 174 paint samples ranged from ND-3,900 ppm. The highest concentration was 3,900 ppm in green paint, followed by 3,850 ppm and 3,550 ppm in green and white paints, respectively. The highest mean concentration of triethylene glycol was 2,720±170 ppm in grey paint, followed by 2,320±81 ppm in white and 2,190±67 ppm in orange paints. The lowest concentration (1,930±110 ppm) was in cream paints. All of the paint colors contained triethylene glycol, except violet paints.

### Propylene glycol

The concentrations of propylene glycol ranged from ND-2,510 ppm. The highest propylene glycol concentration was 2,510 ppm obtained in blue paint, followed by 2,460 ppm, also in blue paint, and 2,350 ppm in white paint. The highest mean concentration of propylene glycol was 1,850±74 ppm in pink paints, followed by 1,840±140 ppm in white and 1,750±100 ppm in cream paints. The lowest mean concentration, 1,230±1.7 ppm, was obtained in brown paints. All the paint colors contained propylene glycol except orange and violet paints.

#### Ethylene glycol

The concentrations of ethylene glycol ranged from ND-2,710 ppm. The highest ethylene glycol concentration was 2,710 ppm in white paint, followed by 2,700 ppm also in white paint and 2,520 ppm in pink paint. The highest mean concentration of ethylene glycol was 2,430±120 ppm in pink paints, followed by 2,280±4.4 ppm in orange paints and 2,220±45 ppm in chocolate paints. The lowest concentration (1,490±170 ppm) was obtained in brown paints. Ethylene glycol was present in all of the paint samples except for grey, red and violet.

Generally, the highest mean concentration of the four anti-freezing agents in all of the 174 paint samples was 2,720±170 ppm, triethylene glycol obtained in grey paints. This was followed by 2,430±120 ppm, ethylene glycol in pink paints and 2,320±81 ppm, triethylene glycol in white paints. The lowest anti-freezing agent concentration was 1,230±1.7 ppm, propylene glycol in brown colored paints. No anti-freezing agents were present in violet paints, and propylene glycol and ethylene glycol were not detected in orange and grey paints, respectively.

### Multivariate statistical analyses

#### Correlation coefficient

The data obtained in the present study were subjected to Pearson's correlation coefficient analysis. Triethylene glycol had a positive correlation with manufacturers versus diethylene glycol, and ethylene glycol versus manufacturers (*Table 7*). There was a possible linear association between manufacturers and diethylene glycol and ethylene glycol. Triethylene glycol and diethylene glycol were found in all of the colors except for violet (*Table 6*).

**Table 7:** Correlation Coefficient of Anti-freezing Agents in Paint Samples

**S/N**	**Manufacturers**	**Colors**	**DEG**	**TEG**	**PG**	**EG**
***Manufacturers***	1	−0.088	−0.106	.281^**^	−0.171	0.414^**^
***Colors***		1	0.052	0.052	0.019	−0.068
***DEG***			1	0.391^**^	−0.124	−0.241^*^
***TEG***				1	−0.400^**^	−0.151
***PG***					1	0.322^**^
***EG***						1

Abbreviations: DEG, diethylene glycol; EG, ethylene glycol; PG, propylene glycol; TEG, triethylene glycol

Note:

^**^ significant at 0.01

^*^significant at 0.05

#### Principal component analysis

One principal component was extracted using PCA, hence there was no rotated component matrix. Corresponding components, variable loadings, and variances are presented in Table 8. Only PCs with eigenvalues greater than 1 were considered. Principal component analysis of the whole data set yielded one data set explaining 45.55% of the total variance. The component was responsible for 45.55% of variance and was best represented by triethylene glycol. Triethylene glycol was extensively used by most of the manufacturers (A, B, D, F and G-M), except for manufacturers C, E and N, despite its potential acute health effects on humans and animals.

**Table 8:** Total Variance and Component Matrix of Anti-freezing Agents

**(a) Total Variance Explained**						

**Component**	**Initial Eigen values**			**Extraction Sums of Squared Loadings**		
	**Total**	**% of Variance**	**Cumulative %**	**Total**	**% of Variance**	**Cumulative %**
1	1.822	45.550	45.550	1.822	45.550	45.550
2	0..913	22.823	68.373			
3	0.822	20.561	88.934			
4	0.443	11.066	100.000			
**(b) Component matrixes**						

	**Compounds**			**Component matrix PC1**		
	TEG			**0.753**		
	PG			−0.698		
	**DEG**			**0.639**		
	EG			−0.600		

Abbreviations: DEG, diethylene glycol; EG, ethylene glycol; PG, propylene glycol; TEG, triethylene glycol

Extraction method: PCA; Rotation method: Varimax with Kaiser normalization; Bold figures indicate absolute values > 0.5 of parameters with strong loading value

#### Cluster analysis

**Figure 4:**
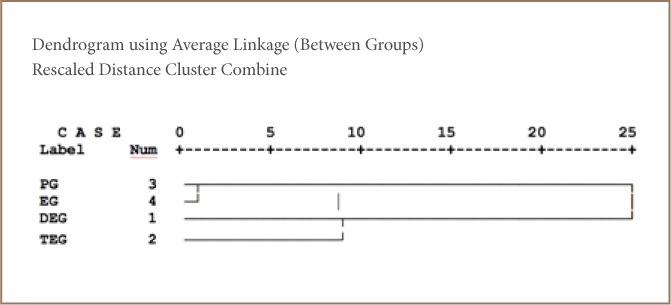
Dendrogram of hierarchical cluster analysis of anti-freezing agents in paint samples

Cluster analysis was performed on the data set using the between-groups linkage method and squared Euclidean distance using hierarchical clustering with Statistical Package for the Social Sciences software. Figure 4 shows the cluster analysis of anti-freezing agents in the paint samples as a dendrogram. Two clusters were obtained. Cluster 1: propylene glycol and ethylene glycol were well correlated with one another and found in the same colors and manufacturers (manufacturers E and N); Cluster 2 was associated with diethylene glycol and triethylene glycol and was found in all of the paint samples except in samples produced by manufacturers G, I, K, N; and C, E and N, respectively. Excessive exposure to their vapors may cause central nervous system depression, metabolic acidosis and nephrotoxicity as nausea, vomiting, abdominal pain, diarrhea, respiratory tract irritation and even death.

## Discussion

The levels of the four anti-freezing agents in the samples in the present study were above the European Union permissible limit of 500 ppm, which came into effect in 2010. The anti-freezing agents concentrations in the 174 samples collected in Lagos and Ibadan are presented in Supplemental Material 1, while Table 9 shows a comparison of the concentrations of anti-freezing agents in the paint samples obtained in this study with a previous study. Out of the 14 manufacturers considered in this study, only 3 manufacturers (manufacturers A, E and N) used propylene glycol in their paint production. Ethylene glycol was only detected in samples from manufacturers E, G, L and N. Triethylene glycol was not detected in any of the paint samples except for those produced by manufacturers C, E (registered manufacturers) and N (unregistered manufacturer). Only manufacturer C did not use any of the 4 anti-freezing agents in its paint production. Manufacturers I and K used only triethylene glycol in their products. Most of the paint manufacturers used between 2 to 3 anti-freezing agents in their products. Variations in the concentrations of compounds found in the paint samples might be attributed to different paint formulations used by manufacturers. Some of the factors which might affect the levels of anti-freezing agents in paint formulations include cost of raw materials, final cost of paint production, availability of raw materials, characteristics of the finished paint products with regard to the different percentages of the raw materials used, acceptability of the different finished paint products by the consumer and others. The order of anti-freezing agent concentrations in paints with respect to manufacturers were:

Diethylene glycol: D > A> M > J > L > B > H > F
Triethylene glycol: G > K > M > L > I > F > J > H > A > B > D
Propylene glycol: N > A > E
Ethylene glycol: L > N > E > G

**Table 9:** Comparison with Available Literature

	**Present study**	**Nakashima et al., 2007**
Number of VOCs determined	4	12
Number of manufacturers studied	14	6
Types of paints used	174 multi-purpose	6 indoor 9 multi-purpose
Analytical procedures	GC-MS, GC-FID	GC-MS, GC-FID
	**Maximum concentration value (ppm)**	**Maximum concentration value (ppm)**
EG	2,700	5,300
DEG	1,980	ND
TEG	3,900	2,000
PG	2,510	ND

Abbreviations: DEG, diethylene glycol; EG, ethylene glycol; PG, propylene glycol; TEG, triethylene glycol

All of the paint colors contained anti-freezing agents except for violet paints. This might be attributed to the pigment used in the color production and the presence of other raw materials in the paints. Ethylene glycol was present in all of the paint colors considered in this study except for grey, red and violet; propylene glycol was present in all of the paint colors except for orange and violet; and triethylene glycol and diethylene glycol were present in all of the paint colors except for violet. The order of anti-freezing agents with respect to paint colors were:

Diethylene glycol: white > grey > chocolate > pink > brown > red > blue = cream = green > yellow > orange; Triethylene glycol: grey > white > orange > yellow > pink > green > brown > chocolate > red > blue > cream;
Propylene glycol: pink > white > cream > green > blue > red > grey = yellow > chocolate > brown;
Ethylene glycol: pink > orange > chocolate > white > blue > green > cream > yellow > brown.

Propylene glycol (1, 2-propanediol) is generally considered safe, but when used in high amounts or for prolonged exposure periods, toxicity can occur. The reported adverse effects of propylene glycol include central nervous system toxicity, hyperosmolarity, hemolysis, cardiac arrhythmia, seizures, agitation, and lactic acidosis.^32^ People at risk of toxicity include infants, those with renal or hepatic insufficiency, epilepsy, and burn patients receiving extensive dermal applications of propylene glycol-containing products.^33^,^34^ The widespread use of ethylene glycol as an anti-freezing agent is based on its ability to lower the freezing point when mixed with water. Ethylene glycol ingestion may cause serious poisoning due to high toxicity.^35^,^36^ It is readily absorbed from the gastrointestinal tract, and the maximum blood concentration is reached within 2-4 hours.^37^ Ethylene glycol toxicity includes central nervous system depression, renal failure and coma.^38–41^ Depending on dose, cardiorespiratory symptoms with elevations in heart rate and blood pressure after 12–24 hours and renal failure after 24 to 72 hours have been reported.^42^ Exposure to triethylene glycol can cause peripheral sensory irritant effect, while repeated exposure to triethylene glycol aerosol may result in respiratory tract irritation with cough, shortness of breath and tightness of the chest.^7^,^43^ Diethylene glycol has been classified as hazardous under the Globally Harmonized System of Classification and Labeling of Chemicals due to its health effects.^44^,^45^ Ingestion may cause central nervous system depression, damage to the digestive tract, lungs, liver, brain, kidney, and pancreas.^46^

## Conclusions

The present study identified VOCs present in 174 paint samples marketed in Lagos and Ibadan, Nigeria and assessed four oxygenated VOCs: ethylene glycol, diethylene glycol, triethylene glycol and propylene glycol present in paint samples. In most cases, the levels of anti-freezing agents in water-based paints sold in Nigeria were above the European Union permissible limits of 500 ppm. Concentrations were higher in products produced by unregistered manufacturers compared to those produced by registered manufacturers. As reported by the European Union, ethylene glycol and diethylene glycol are prohibited anti-freezing agents.^9^ Results of the multivariate statistical analysis clearly showed that triethylene glycol was the commonly used anti-freezing agent, and had the highest concentration of 3,900 ppm in green paints produced by an unregistered manufacturer, manufacturer G. Presently, there are no national regulations in place to control the use of these compounds in paints. In addition, there are no indications of solvents or other raw materials on paint labels. Volatile organic compounds and other raw materials in consumer products such as paints requiring caution should be indicated clearly on product labels. An assessment of toxic compounds in paints marketed in Nigeria and their emissions is needed. There is also a need for stringent regulations to safeguard public health from occupational exposures to toxic and prohibited compounds in paints.
