# Supplementary material for: Oxygenated Volatile Organic Compounds (Anti-freezing Agents) in Decorative Water-based Paints Marketed in Nigeria
Source: J Health Pollut. 2018 Jun 11;8(18):180606. doi: 10.5696/2156-9614-8.18.180606 (PMC6239063; doi:10.5696/2156-9614-8.18.180606)
Supplement: Supplementary file 1 [file hapn-8-18-180606_s01.docx]

|  | **Supplemental Material**  **Concentrations (ppm) of Anti-freezing Agents in Paint  Samples with Respect to Manufacturers** | | | | | | | | | | | |
| --- | --- | --- | --- | --- | --- | --- | --- | --- | --- | --- | --- | --- |
|  |  |  | **DEG** | | **TEG** | | **PG** | | **EG** | |  |  |
| **S/N** | **Manufacturer**  **codes** | **Primary colors in paint samples** | **Sample 1** | **Sample 2** | **Sample 1** | **Sample 2** | **Sample 1** | **Sample 2** | **Sample 1** | **Sample 2** | |  |
| 1 | A | Blue | 1420 | 1520 | 1610 | 1850 | 1170 | 1090 | ND | ND | |  |
|  |  | Brown | 1250 | 1310 | 1690 | 1940 | 1230 | 1230 | ND | ND | |  |
|  |  | Chocolate | 1840 | 1670 | 1220 | 1120 | 1450 | 1510 | ND | ND | |  |
|  |  | Cream | 1840 | 1910 | 1510 | 1070 | 1830 | 1570 | ND | ND | |  |
|  |  | Green | 1120 | 1250 | 1870 | 1700 | 1720 | 1450 | ND | ND | |  |
|  |  | Grey | 1730 | 1680 | 2840 | 2600 | 1750 | 1450 | ND | ND | |  |
|  |  | Pink | 1610 | 1480 | 2030 | 1960 | 1900 | 1800 | ND | ND | |  |
|  |  | Red | 1190 | 1340 | 1520 | 1910 | 1640 | 1610 | ND | ND | |  |
|  |  | White | 1020 | 1270 | 1550 | 1260 | 1990 | 1590 | ND | ND | |  |
|  |  | Yellow | 1440 | 1250 | 2670 | 2190 | 1810 | 1770 | ND | ND | |  |
| 2 | B | Blue | 1480 | 1310 | 1630 | 1600 | ND | ND | ND | ND | |  |
|  |  | Green | 1190 | 1240 | 1840 | 1870 | ND | ND | ND | ND | |  |
|  |  | Orange | 1250 | 1350 | 1900 | 1980 | ND | ND | ND | ND | |  |
|  |  | Pink | 1570 | 1500 | 1520 | 1480 | ND | ND | ND | ND | |  |
|  |  | Red | 1650 | 1050 | 1520 | 1600 | ND | ND | ND | ND | |  |
|  |  | White | 1400 | 1180 | 1710 | 1830 | ND | ND | ND | ND | |  |
|  |  | Yellow | 1510 | 1520 | 1480 | 1500 | ND | ND | ND | ND | |  |
|  |  | Brown | 1420 | 1520 | 1860 | 1930 | ND | ND | ND | ND | |  |
|  |  | Cream | 1230 | 1120 | 1600 | 1450 | ND | ND | ND | ND | |  |
| 3 | C | Blue | ND | ND | ND | ND | ND | ND | ND | ND | |  |
|  |  | Brown | ND | ND | ND | ND | ND | ND | ND | ND | |  |
|  |  | Chocolate | ND | ND | ND | ND | ND | ND | ND | ND | |  |
|  |  | Green | ND | ND | ND | ND | ND | ND | ND | ND | |  |
|  |  | Grey | ND | ND | ND | ND | ND | ND | ND | ND | |  |
|  |  | Red | ND | ND | ND | ND | ND | ND | ND | ND | |  |
|  |  | Violet | ND | ND | ND | ND | ND | ND | ND | ND | |  |
|  |  | White | ND | ND | ND | ND | ND | ND | ND | ND | |  |
|  |  | Yellow | ND | ND | ND | ND | ND | ND | ND | ND | |  |
| 4 | D | Blue | 1250 | 1560 | 1830 | 1720 | ND | ND | ND | ND | |  |
|  |  | Cream | 1520 | 1240 | 1740 | 1770 | ND | ND | ND | ND | |  |
|  |  | Green | 1560 | 1600 | 1000 | 1110 | ND | ND | ND | ND | |  |
|  |  | Red | 1800 | 1850 | 1300 | 1130 | ND | ND | ND | ND | |  |
|  |  | White | 1800 | 1700 | 2130 | 2230 | ND | ND | ND | ND | |  |

|  | **Supplementary material contd’: Concentrations (ppm) of anti-freezing agents in paint samples with respect to manufacturers** | | | | | | | | | | |
| --- | --- | --- | --- | --- | --- | --- | --- | --- | --- | --- | --- |
|  |  |  | **DEG** | | **TEG** | | **PG** | | **EG** | |  |
| S/N | Manufacturer  codes | **Primary colors in paint samples** | **Sample 1** | **Sample 2** | **Sample 1** | **Sample 2** | **Sample 1** | **Sample 2** | **Sample 1** | **Sample 2** |  |
| 5 | E | Blue | ND | ND | ND | ND | 1380 | 1400 | 1960 | 2000 |  |
|  |  | Cream | ND | ND | ND | ND | 1840 | 1870 | 2000 | 2190 |  |
|  |  | Green | ND | ND | ND | ND | 1400 | 1490 | 1370 | 1500 |  |
|  |  | White | ND | ND | ND | ND | 1460 | 1460 | 2280 | 2300 |  |
|  |  | Yellow | ND | ND | ND | ND | 1500 | 1320 | 1500 | 1510 |  |
| 6 | F | Blue | 1080 | 1170 | 1620 | 1510 | ND | ND | ND | ND |  |
|  |  | Cream | 1140 | 1070 | 1920 | 1890 | ND | ND | ND | ND |  |
|  |  | Green | 1000 | 1100 | 1930 | 1820 | ND | ND | ND | ND |  |
|  |  | Red | 1140 | 1140 | 1820 | 1840 | ND | ND | ND | ND |  |
|  |  | White | 1720 | 1980 | 2630 | 2920 | ND | ND | ND | ND |  |
|  |  | Yellow | 1120 | 1240 | 2300 | 2540 | ND | ND | ND | ND |  |
| 7 | G | Blue | ND | ND | 2160 | 2000 | ND | ND | 1490 | 1540 |  |
|  |  | Cream | ND | ND | 2520 | 2980 | ND | ND | 1350 | 1580 |  |
|  |  | Green | ND | ND | 3850 | 3900 | ND | ND | 2000 | 1860 |  |
|  |  | White | ND | ND | 2570 | 2750 | ND | ND | 1430 | 1450 |  |
| 8 | H | Blue | 1200 | 1200 | 1770 | 1670 | ND | ND | ND | ND |  |
|  |  | Cream | 1110 | 1240 | 1750 | 1630 | ND | ND | ND | ND |  |
|  |  | Green | 1290 | 1230 | 1180 | 1160 | ND | ND | ND | ND |  |
|  |  | Pink | 1580 | 1550 | 2240 | 2090 | ND | ND | ND | ND |  |
|  |  | White | 1430 | 1540 | 2370 | 2600 | ND | ND | ND | ND |  |
| 9 | I | Blue | ND | ND | 2310 | 2430 | ND | ND | ND | ND |  |
|  |  | Cream | ND | ND | 2140 | 2270 | ND | ND | ND | ND |  |
|  |  | Green | ND | ND | 1930 | 1730 | ND | ND | ND | ND |  |
|  |  | White | ND | ND | 2480 | 2230 | ND | ND | ND | ND |  |
| 10 | J | Blue | 1230 | 1380 | 1840 | 1920 | ND | ND | ND | ND |  |
|  |  | Cream | 1230 | 1400 | 2000 | 2030 | ND | ND | ND | ND |  |
|  |  | Green | 1680 | 1620 | 1200 | 1290 | ND | ND | ND | ND |  |
|  |  | Red | 1480 | 1620 | 2430 | 2400 | ND | ND | ND | ND |  |
|  |  | White | 1200 | 1320 | 2160 | 2460 | ND | ND | ND | ND |  |
| 11 | K | Blue | ND | ND | 2850 | 2800 | ND | ND | ND | ND |  |

|  |  | | | | | | | | | | |
| --- | --- | --- | --- | --- | --- | --- | --- | --- | --- | --- | --- |
|  | **Supplementary material contd’: Concentrations (ppm) of anti-freezing agents in paint samples with respect to manufacturers** | | | | | | | | | |  |
|  |  |  | **DEG** | | **TEG** | | **PG** | | **EG** | |  |
| **S/N** | **Manufacturer**  **codes** | **Primary colors in paint samples** | **Sample 1** | **Sample 2** | **Sample 1** | **Sample 2** | **Sample 1** | **Sample 2** | **Sample 1** | **Sample 2** |  |
| 11 | K | Chocolate | ND | ND | 2360 | 2220 | ND | ND | ND | ND |  |
|  |  | Cream | ND | ND | 2330 | 2430 | ND | ND | ND | ND |  |
|  |  | Green | ND | ND | 2530 | 2830 | ND | ND | ND | ND |  |
|  |  | Red | ND | ND | 2640 | 2410 | ND | ND | ND | ND |  |
|  |  | White | ND | ND | 3140 | 3550 | ND | ND | ND | ND |  |
| 12 | L | Blue | 1160 | 1520 | 2170 | 2620 | ND | ND | 2100 | 2450 |  |
|  |  | Brown | 1630 | 1240 | 2160 | 2290 | ND | ND | 1440 | 1540 |  |
|  |  | Chocolate | 1300 | 1310 | 2330 | 2270 | ND | ND | 2180 | 2250 |  |
|  |  | Cream | 1570 | 1320 | 1700 | 1910 | ND | ND | 1930 | 1920 |  |
|  |  | Green | 1290 | 1410 | 2430 | 2160 | ND | ND | 2460 | 2450 |  |
|  |  | Orange | 1280 | 1330 | 2170 | 2160 | ND | ND | 2290 | 2280 |  |
|  |  | Pink | 1490 | 1650 | 2280 | 2390 | ND | ND | 2340 | 2520 |  |
|  |  | White | 1320 | 1310 | 2130 | 2230 | ND | ND | 2700 | 2710 |  |
| 13 | M | Blue | 1790 | 1460 | 1720 | 1310 | ND | ND | ND | ND |  |
|  |  | Cream | 1340 | 1490 | 2060 | 1910 | ND | ND | ND | ND |  |
|  |  | Green | 1710 | 1540 | 2820 | 3110 | ND | ND | ND | ND |  |
|  |  | Orange | 1410 | 1080 | 2350 | 2550 | ND | ND | ND | ND |  |
|  |  | Pink | 1120 | 1490 | 2590 | 2470 | ND | ND | ND | ND |  |
|  |  | Red | 1010 | 1200 | 2440 | 2470 | ND | ND | ND | ND |  |
|  |  | White | 1880 | 1570 | 2050 | 2030 | ND | ND | ND | ND |  |
| 14 | N | Blue | ND | ND | ND | ND | 2510 | 2460 | 2280 | 2380 |  |
|  |  | Cream | ND | ND | ND | ND | 1710 | 1700 | 2070 | 2180 |  |
|  |  | Green | ND | ND | ND | ND | 2140 | 2190 | 2180 | 2170 |  |
|  |  | White | ND | ND | ND | ND | 2210 | 2350 | 2080 | 2110 |  |

Abbreviations: ND, not detected
